# DM-MOGA: a multi-objective optimization genetic algorithm for identifying disease modules of non-small cell lung cancer

**DOI:** 10.1186/s12859-023-05136-z

**Published:** 2023-01-09

**Authors:** Junliang Shang, Xuhui Zhu, Yan Sun, Feng Li, Xiangzhen Kong, Jin-Xing Liu

**Affiliations:** School of Computer Science, Qufu Normal University, Rizhao, 276826 China

**Keywords:** Disease module identification, Biological network construction, Gene expression data, Genetic algorithm, Multi-objective optimization

## Abstract

**Background:**

Constructing molecular interaction networks from microarray data and then identifying disease module biomarkers can provide insight into the underlying pathogenic mechanisms of non-small cell lung cancer. A promising approach for identifying disease modules in the network is community detection.

**Results:**

In order to identify disease modules from gene co-expression networks, a community detection method is proposed based on multi-objective optimization genetic algorithm with decomposition. The method is named DM-MOGA and possesses two highlights. First, the boundary correction strategy is designed for the modules obtained in the process of local module detection and pre-simplification. Second, during the evolution, we introduce Davies–Bouldin index and clustering coefficient as fitness functions which are improved and migrated to weighted networks. In order to identify modules that are more relevant to diseases, the above strategies are designed to consider the network topology of genes and the strength of connections with other genes at the same time. Experimental results of different gene expression datasets of non-small cell lung cancer demonstrate that the core modules obtained by DM-MOGA are more effective than those obtained by several other advanced module identification methods.

**Conclusions:**

The proposed method identifies disease-relevant modules by optimizing two novel fitness functions to simultaneously consider the local topology of each gene and its connection strength with other genes. The association of the identified core modules with lung cancer has been confirmed by pathway and gene ontology enrichment analysis.

**Supplementary Information:**

The online version contains supplementary material available at 10.1186/s12859-023-05136-z.

## Background

Lung cancer is the cancer with the highest mortality rate worldwide, and about 80% of cases are non-small cell lung cancer (NSCLC) that has a poor 5-year survival rate (average, 9–11 months) [1]. In recent years, research on molecular mechanisms of lung cancer has promoted the development of their corresponding targeted drugs which have greatly improved the survival and prognosis of patients [2]. Meanwhile, more and more evidence has indicated that a group of genes related to a specific disease do not work in isolation, on the contrary, they usually interact with each other, thus gene co-expression networks (GCNs) have become a competitive model. Analysis on GCNs can help researchers identify disease modules. A disease module is considered as a subnetwork that contains most of the disease-related genes with compact topological connections and closely related functions, providing a system-level understanding of disease pathogenesis [3].

In recent years, many methods have been proposed for identifying disease modules. In general, these methods can be divided into four categories: local expansion, machine learning, mathematical programming and evolutionary algorithm (EA) [4]. DISeAse MOdule Detection algorithm (DIAMOnD) is a typical method based on local expansion which uses known disease-associated genes (seeds) to iteratively expand modules by evaluating the significance of the number of connections between seeds and other genes [5]. Although this method can identify disease modules, the coverage of disease-related genes may be low. The node and edge Prioritization-based Community Analysis is a knowledge-guided and network-based integration method to reveal functional modules in non-small cell lung cancer [6]. The protein–protein interaction network is prioritized by a random walk algorithm based on NSCLC seed genes and integrating edge weights, and then a "community network" is constructed in combination with Girvan-Newman and Label Propagation algorithms. MTGO is another method for functional module detection based on prior biological knowledge and topology information [7]. It directly utilizes gene ontology (GO) terms during module detection and labels each module with the most appropriate GO term, thereby simplifying the functional interpretation of modules. Molecular Complex Detection (MCODE) is a popular method without using prior knowledge for seeds [8]. It calculates the weight for each vertex according to the local neighborhood density. Nodes with the largest weight are selected as seeds, then the method traverses outwards and incorporates nodes with the weight higher than a given threshold into the module. SWItch Miner (SWIM) is a method to identify small modules containing key regulatory genes (switch genes) by introducing three topological attribute statistics for nodes [9]. SWIM was applied to a dataset from The Cancer Genome Atlas (TCGA) to characterize the etiology of interesting diseases. Identifying disease modules through machine learning methods is another efficient way. Wu and Stein used Markov Clustering (MCL) to cluster the weighted gene functional interaction network into a series of disease modules to respectively identify prognostic biomarkers of breast cancer and ovarian cancer [10]. PS-MCL (Parallel Shotgun Coarsened MCL) was proposed by Lim et al*.*, a parallel community detection method that outperforms MCL in both runtime and the division quality [11]. PS-MCL adopts an effective coarsening scheme called shotgun coarsening (SC) to improve the module fragmentation problem of MCL, while providing a multi-core parallel algorithm for community detection to increase scalability. In addition, machine learning based methods are more efficient to identify disease modules from multi-omics data. A greedy decision forest is proposed to identify community structure from molecular interaction networks [12]. It obtains a high degree of interpretability by using shapley additive explanations. A strongly interconnected disease module identification method called SigMod is proposed by *Liu *et al*.* based on mathematical programming [13]. It identifies disease modules by integrating the results of genome-wide association study (GWAS) and gene networks, as well as optimizing the binary quadratic objective function by a graph min-cut approach. EAs are popular and widely used in the field of disease module identification. Multi-objective evolutionary algorithm (MOEA) DiffCoMO identifies differential co-expression modules by maximizing the difference between module membership value of genes corresponding to two different infection stages [14]. A new method ModuleDiscoverer is proposed to identify regulatory modules from the protein–protein interaction network (PPIN) and gene expression data [15]. It uses a randomization heuristic-based approximation of community structure to discover modules according to the maximum clique enumeration problem.

In this research, we construct a GCN relying on the PPIN, then we develop a disease-related module identification method based on the multi-objective genetic algorithm, named DM-MOGA. This method is utilized to analyze the obtained network by optimizing two fitness functions which can evaluate the functional similarity and the density of the topological connection of modules, respectively. In addition, a boundary correction strategy is designed for local modules obtained by pre-simplification, to reconfirm the genes in the margin belonging to which module.

## Methods

### Network construction

Studies have confirmed that integrating gene expression data and PPIN helps people understand the complex multi-layered molecular structure of human diseases. Therefore, two gene expression datasets of NSCLC in the NCBI Gene Expression Omnibus (GEO) database are selected to construct GCNs with PPIN information being referred to, respectively. Detailed steps of data preprocessing and network construction are as follows. First of all, limma package in the R/Bioconductor software is utilized to identify differentially expressed genes (DEGs) whose t-statistics *p* value are adjusted by the Benjamini–Hochberg method [16]. Genes with the adjusted *p* value less than 0.05 are considered as DEGs. Only interactions between DEGs are used to construct GCNs.

To estimate the interaction intensity between DEGs, a new criterion called Gaussian Copula Mutual Information (GCMI) is introduced [17]. GCMI uses the concept of a statistical copula to provide the advantages of Gaussian parametric estimation for variables with any type of marginal distributions, and it is suitable for estimating MI between two continuous variables. At the same time, we use the $sim_{Rel}$ score to calculate and compare functionally related products of a pair of DEGs which provides a similarity criterion for gene ontology (GO) terms of two gene products. The computation of $sim_{Rel}$ has been implemented by the R package GOSemSim [18]. The definition of $sim_{Rel}$ is as follows,

1 $$sim_{Rel} \left( {c_{1} ,c_{2} } \right) = \mathop {\max }\limits_{{c \in S\left( {c_{1} ,c_{2} } \right)}} \left( {\frac{2 \cdot \log p\left( c \right)}{{\log p\left( {c_{1} } \right) + \log p\left( {c_{2} } \right)}} \cdot \left( {1 - p\left( c \right)} \right)} \right)$$

where $S\left( {c_{1} ,c_{2} } \right)$ is the set of common ancestors of GO terms $c_{1}$ and $c_{2}$, $p\left( c \right)$ is the probability of $c$. It is utilized to calculate a fitness value in "Fitness functions" section.

After obtaining the correlation matrix between DEGs, it is compared with the PPIN. We downloaded the PPIN from the human protein reference database (HPRD) which contains 39,240 interactions [19]. The original protein–protein interaction information is presented in Additional file 1. If a correlation does not exist in the PPIN, it will be modified to 0.

### DM-MOGA framework

**Fig. 1:**
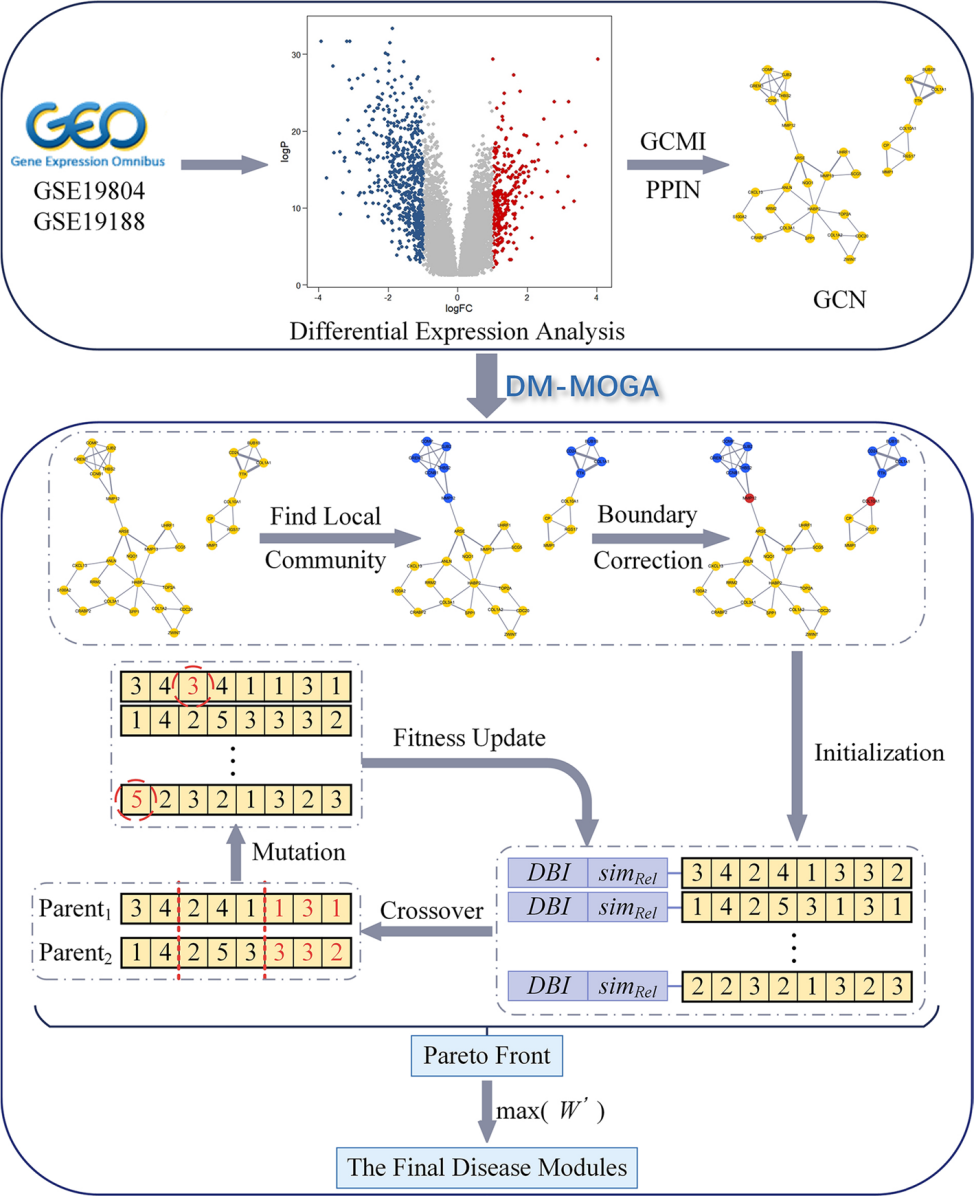
The DM-MOGA workflow

Due to the high complexity of identifying disease modules from a large-scale GCN, heuristic strategies are required to guide the search process. One of the most popular strategies is EA which are suitable for solving global optimization problems in the discrete search space [20]. EA is an optimization algorithm inspired by Darwin's principles of natural selection. Each solution is described as an individual in the population, and each individual is associated with one or more fitness functions optimized by natural selection process. In this paper, we propose a new method DM-MOGA based on MOEA and decomposition to identify modules which is regarded as biomarkers of NSCLC. The workflow of DM-MOGA is displayed in Fig. 1. In this section, we describe the framework of DM-MOGA, including pre-simplification with boundary correction, chromosome encoding and initialization scheme, operators of MOEA and the optimal solution selection strategy. After the evolution is completed, the result with the largest $W^{\prime}$ in the Pareto front is considered as the final solution that contains hundreds of modules. We only select the biggest module involving more biological information from the GCN.

#### Local module pre-simplification and boundary correction

In order to improve the adaptability of DM-MOGA for large-scale biological networks, a pre-simplification strategy for local module (LM) is introduced from [21] and executed before the evolution. This strategy randomly selects one node $a$ from the network, then a LM is defined as containing node $a$, its neighbor $a_{k}$ with the largest degree, $a_{k}^{\prime } s$ neighbor $a_{kk}$ that has the largest number of common neighbors with $a_{k}$, and all joint neighbors of $a_{k}$ and $a_{kk}$. For neighbors of all nodes in the LM, those neighbors whose number of connections with LM is beyond half of its degree are also added to LM. Finally, a complete graph of order 3 in the LM is selected and simplified to a single node. Specifically, we will not consider the possibility that nodes in the LM do not belong to the same module during the evolution. Above operations are repeated to find another LM from the remaining nodes of the network until all nodes are assigned to a LM.

However, this strategy only considers the topology of the network without the weight of edges, and vertices of some edges with smaller weights do not necessarily belong to a LM. Therefore, we develop a module boundary correction strategy. In this strategy, the matrix $NodeTable$ for all nodes is maintained, of which each row represents a node. The first column is the index of the node, the second column is the module to which the node belongs after the LM pre-simplification strategy, and the third column is the new module to which the node belongs after the boundary correction.

The constructed GCN is denoted as $G = \left( {V,E} \right)$, where $V = \left\{ {v_{i} \left| {i = 1,2, \ldots ,N} \right.} \right\}$ is the set of nodes, $E = \left\{ {e_{i} \left| {i = 1,2, \ldots ,M} \right.} \right\}$ is the set of connections between a pair of nodes. Firstly, calculate the weight $\{ V_{1}^{w} ,V_{2}^{w} , \ldots ,V_{i}^{w} , \ldots V_{N}^{w} \}$ for each node in the network. Specifically, the most densely connected area in the module made up of node $i$ and its immediate neighbors is defined as the highest *k*-core, and $V_{i}^{w}$ is the product of the density and the minimum degree of the highest k-core. The density of the highest *k*-core is $D^{G} = \frac{2E}{{\left| V \right|\left( {\left| V \right| - 1} \right)}}$ [8]. Then, we get the attribute $\{ V_{1}^{a} ,V_{2}^{a} , \ldots ,V_{i}^{a} , \ldots V_{N}^{a} \}$ for each node, where $V_{i}^{a} = \sum\nolimits_{j} {E_{ij} }$, $j \in LC_{{NodeTable\left( {i,2} \right)}}$. For each module, if node $i$ satisfies $(V_{i}^{w} > 2)\& (V_{i}^{w} \ge \overline{{V^{w} }} )$, node $i$ is reserved in this module; otherwise, the third element of the corresponding row of node $i$ in the $NodeTable$ is set to 0, indicating that node $i$ will be reassigned afterwards. Secondly, for each LM, the node with the largest sum of the weight of connecting edges is selected as the seed node, and neighbors of this seed are also recursively assigned to this module. For those nodes that still cannot be allocated to a LM, they are retained in the network independently.

#### Fitness functions

The proposed DM-MOGA identifies disease modules by minimizing the following two fitness functions. The first function is Davies–Bouldin Index (DBI) which should have been a measure to evaluate the quality of clustering results [22]. The basic idea of DBI is to evaluate the distance between two clusters, considering that the distance between nodes belonging to different clusters should be as large as possible and the distance between nodes within a cluster should be as small as possible. With the similarity matrix obtained by calculating $sim_{Rel}$ between genes, DBI is applied to assess the similarity of functions of genes belonging to the same disease module. The formula is as follows:

2 $$DBI = \frac{1}{{C_{num} }}\sum\limits_{i = 1}^{{C_{num} }} {\mathop {\max }\limits_{i;j \ne i} \frac{{S_{i} + S_{j} }}{{dist\left( {v_{i} ,v_{j} } \right)}}}$$

where $C_{num}$ is the number of modules, $dist\left( \cdot \right)$ is the $sim_{Rel}$ similarity between two nodes, $S_{i} = \frac{1}{{\left| {C_{i} } \right|}}\sum\nolimits_{{x_{j} \in C_{i} }} {dist\left( {x_{j} ,v_{i} } \right)}$ measures the extent of dispersion of module $C_{i}$, $C_{i}$ represents the *i*-th module, $x_{j}$ is the *j*-th node of $C_{i}$, and $v_{i}$ represents the center of $C_{i}$.

The second function is a modified clustering coefficient ($CC^{\prime}$) suitable for weighted networks. In general, $CC^{\prime}$ quantifies the aggregation of one node and its neighbors, but it is modified to the sum of $CC^{\prime}$ of nodes to evaluate the overall module, and the specific formula is as follows. For a random module $C_{i}$, clustering coefficient is defined as:

3 $$CC_{i}^{\prime } = \sum\nolimits_{{x_{j} \in C_{i} }} {\frac{{2\sum\nolimits_{{x_{l} ,x_{k} \in R_{{x_{j} }} \wedge l \ne k}} {E\left( {x_{l} ,x_{k} } \right)} }}{{\left| {R_{{x_{j} }} } \right|\left( {\left| {R_{{x_{j} }} } \right| - 1} \right)}}}$$

where $R_{{x_{j} }}$ is the set of neighbors of node $x_{j}$. Since the result of MOEA is a group of modules, the second fitness function is set to the maximum of $CC^{\prime}$.

The criterion $W$ proposed by Zhao et al*.* is used to select a solution from the Pareto front obtained by MOEA as the final result [23]. The result with the largest $W$ is considered as the final result. The original $W$ is applied to extract a module from unweighted social networks. In order to put all modules into consideration and adapt to weighted GCNs, $W$ is changed to the following form:

4 $$W^{\prime} = \sum\limits_{i = 1}^{{C_{num} }} {\left( {\frac{{O\left( {C_{i} } \right)}}{{\left| {C_{i} } \right|^{2} }} - \frac{{B\left( {C_{i} } \right)}}{{\left| {C_{i} } \right|\left| {C_{i}^{^{\prime}} } \right|}}} \right)}$$

where $C_{i}^{\prime }$ is the complement of $C_{i}$, $O\left( {C_{i} } \right) = \sum\limits_{{j,k \in C_{i} }} {E_{j,k} }$, $B\left( {C_{i} } \right) = \sum\limits_{{j \in C_{i} ,k \in C_{i}^{^{\prime}} }} {E_{j,k} }$.

#### Multi-objective optimization based on decomposition

The basic theory is multi-optimization problem (MOP) based on Pareto optimum which is to optimize a group of functions at the same time:

5 $$\min F\left( x \right) = \left( {f_{1} \left( x \right),f_{2} \left( x \right), \ldots ,f_{k} \left( x \right)} \right)^{T}$$

where $x = \left[ {x_{1} ,x_{2} , \ldots ,x_{N} } \right] \in \Omega$ and $\Omega$ is the feasible region. Then, the definition of dominance relationship is explained, that is, $x_{A}$ dominates $x_{B}$ (written as $x_{A} \succ x_{B}$, $x_{A} ,x_{B} \in \Omega$) if and only if:

6 $$\forall i \in \left\{ {1,2, \ldots ,k} \right\}\;f_{i} \left( {x_{A} } \right) \le f_{i} \left( {x_{B} } \right) \wedge \exists j \in \left\{ {1,2, \ldots ,k} \right\}\;f_{j} \left( {x_{A} } \right) < f_{j} \left( {x_{B} } \right)$$

If there is no vector $x \in \Omega$ such that $x \succ x^{*}$, $x^{*}$ is called a non-dominated solution or Pareto-optimal solution.

MOEA/D-Net is a community detection method based on MOEA with decomposition. It decomposes a MOP into a number of scalar optimization subproblems and optimizes them simultaneously by population evolution. At each iteration, the population is made up of the best solution found for each subproblem since the beginning of evolution. In MOEA/D-Net, the popular Tchebycheff method is used to construct the aggregation function and therefore the scalar optimization subproblems are in the form:

7 $$\min g^{te} \left( {\left. x \right|\lambda_{i} ,z^{*} } \right) = \mathop {\max }\limits_{j = 1}^{2} \left\{ {\lambda_{i}^{j} \left| {F_{j} \left( x \right) - z^{*} } \right|} \right\}$$

where $\lambda = \left\{ {\lambda_{1} ,\lambda_{2} , \ldots ,\lambda_{pop} } \right\}$ is a series of weight vectors uniformly distributed on $\lambda_{i}^{1} + \lambda_{i}^{2} = 1$, $\lambda = \left\langle {\lambda_{i}^{1} ,\lambda_{i}^{2} } \right\rangle \in \left[ {0,1} \right]$, $i = \left\{ {1,2, \ldots ,pop} \right\}$, $pop$ is the population size, and $z^{*} = \left\langle {z_{1}^{*} ,z_{2}^{*} } \right\rangle$ is the reference point in which each point $z_{j}^{*}$ corresponds to the minimum value of a fitness function obtained from the population. For each target vector $\lambda_{i}$, calculate the Euclidean distance between all weight vectors and $\lambda_{i}$, and the neighborhood of $\lambda_{i}$, denoted as $Neib_{i}$, is made up of $nm$ individuals with the smallest Euclidean distance to $\lambda_{i}$, where $nm$ is a predefined parameter. For each non-dominated individual, there is a weight vector that makes it the optimal solution of Eq. 7, and each optimal solution of Eq. 7 is a Pareto-optimal solution of Eq. 5.

#### Initialization

Individuals are encoded and initialized based on the locus-based adjacency encoding schema which is popular in EA-based community detection algorithms [24]. In an individual, each element is initialized as a random neighbor index of its corresponding node or the index of the corresponding node itself, and then this element is recursively replaced by the index that most neighbors of this node share until the element is not changed. Another variable that needs to be initialized is the reference point $z^{*}$, and it is set to the minimum of two fitness functions in the initial population.

#### The main loop of DM-MOGA

DM-MOGA adopts the similar framework with MOEA/D-Net that is proposed by Gong et al*.* [25]. In this method, the following procedure is applied to evolve the population. Every individual $p_{j} \left( {1 \le j \le pop} \right)$ is used to perform the crossover and mutation operation with another randomly selected individual to generate a $child$. If the Tchebycheff value of the $child$ is better than a neighbor in $Neib_{j}$, replace that neighbor with the $child$ and update the reference point $z^{*}$. Specifically, we choose the two-point crossover to take advantage of protecting the effective connection between nodes. We randomly select two elements $i$ and $j$ (i.e., $1 \le i \le j \le N$), and elements in $\left[ {i,j} \right]$ are exchanged between two parents in the population. After the crossover operation is finished, an individual $p_{j}$ is randomly selected for mutation, on which the neighbor-based mutation is performed. According to the encoding strategy, the mutation operator is to randomly select an element $e_{l}$ in the individual $p_{i}$ and replace the neighbor index in it with the index of other neighbors of the node corresponding to $e_{l}$. DM-MOGA will continue to evolve until the maximum number of generations is reached.

## Results and discussion

### Datasets

In the experiments, two NSCLC expression microarray datasets obtained using the same platform (GPL570) were downloaded from the GEO database. The first dataset (ID: GSE19804) [26] is balanced and contains 60 disease and control samples, respectively. The second dataset (ID: GSE19188) [27] contains 91 disease samples and 65 control samples. In the above two datasets, each sample contains the expression data of 21,879 genes. After differential expression analysis, 7669 DEGs and 10,496 DEGs were respectively selected from GSE19804 and GSE19188. Detailed information of these two datasets is shown in Table 1.

**Table 1:** Details of two datasets

Datasets	Tumor samples	Normal samples	Genes
GSE19804	60	60	21879
GSE19188	91	65	21879

### Comparison with other methods

#### Ground-truth dataset

We integrated four kinds of lung cancer-related genes obtained from the MalaCards database as ground truth, including differentially expressed genes, genes related to lung cancer, genes contained in lung cancer related pathways, top affiliated genes of GO terms related to lung cancer [28].

#### Comparison methods

In the experiment, five methods were used to compare the performance with DM-MOGA, that is, a network reduction-based MOEA for community (module) detection (RMOEA), a disease module identification method SigMod, MCODE, and two classic module identification methods from the R package igraph, that is, Hierarchical Clustering [29] and Louvain [30].

To make a fair comparison, parameters in DM-MOGA and RMOEA were set to the same value, namely, the number of iterations $max\_gen = 100$, the population size $pop = 50$, the neighborhood size $nm = 40$, and the mutation rate was 0.1. In addition, to ensure that SigMod can search for modules smoothly, the parameter $maxjump$ was set to 27, and we used the default value for parameters of other methods.

#### Classification performance of disease and healthy samples

**Fig. 2:**
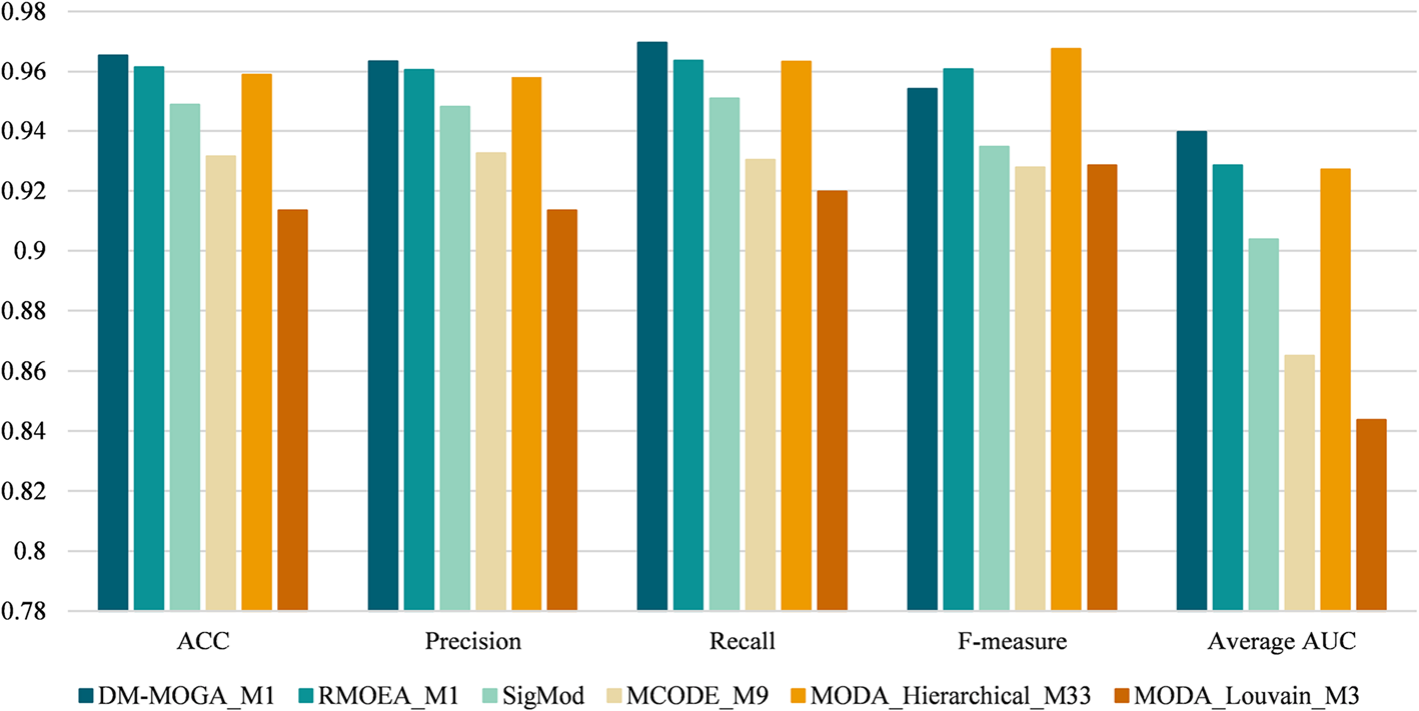
The value of the five-fold cross-validated classification index of the optimal module in GSE19804

**Fig. 3:**
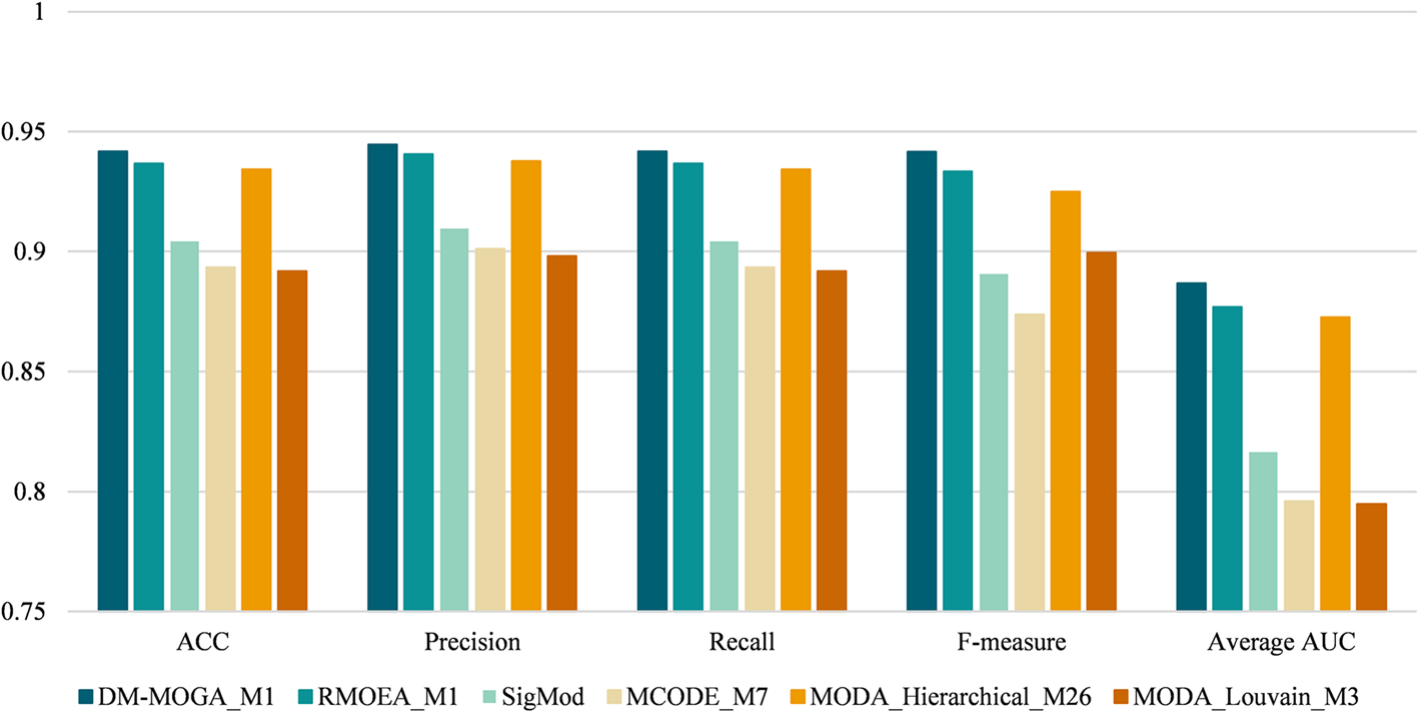
The value of the five-fold cross-validated classification index of the optimal module in GSE19188

Fivefold cross-validation was applied on the largest module to verify its effectiveness as a biomarker. The set of samples is randomly divided into five parts with the same size, one of which is selected as the test set each time, and the other four parts are used for training (the train set). Support vector machine (SVM) is used as the classifier, and the value of five criteria (Accuracy, Precision, Recall, F1 and AUC) of each experiment is taken as the cross-validation result. Since there is a random value during the five-fold cross-validation, for each identified module, we performed five-fold cross-validation for ten times independently, and the final result was the average value of each criterion in experiments. Figures 2 and 3 respectively display the classification performance of the disease module identified by six methods on two NSCLC gene expression datasets. The serial number of the disease module is marked after the abbreviation of comparison methods. According to the figures, module 1 (M1) in community detection results of DM-MOGA obtained the best classification performance on the GSE19804 dataset whose value of five criteria is better than other methods. As for the GSE19188 dataset, the classification performance of the module detected by DM-MOGA was also significant which obtained the maximum value on four of the five metrics. Therefore, the effectiveness of the module obtained from DM-MOGA is verified in guiding the classification of disease and control samples. Moreover, the basic framework of RMOEA is similar to that of DM-MOGA, and both have the ability to effectively guide sample classification. The difference between them is that RMOEA lacks the boundary correction strategy which may lead to unstable results in ten independent experiments. The reason for SigMod, hierarchical clustering and the Louvain algorithm that fails to provide reasonable results might be the same, that is, the default values of their key parameters are not applicable for GCNs. MCODE tends to obtain smaller modules because it has strict conditions for expanding nodes in modules.

#### Enrichment analysis for the disease module

**Fig. 4:**
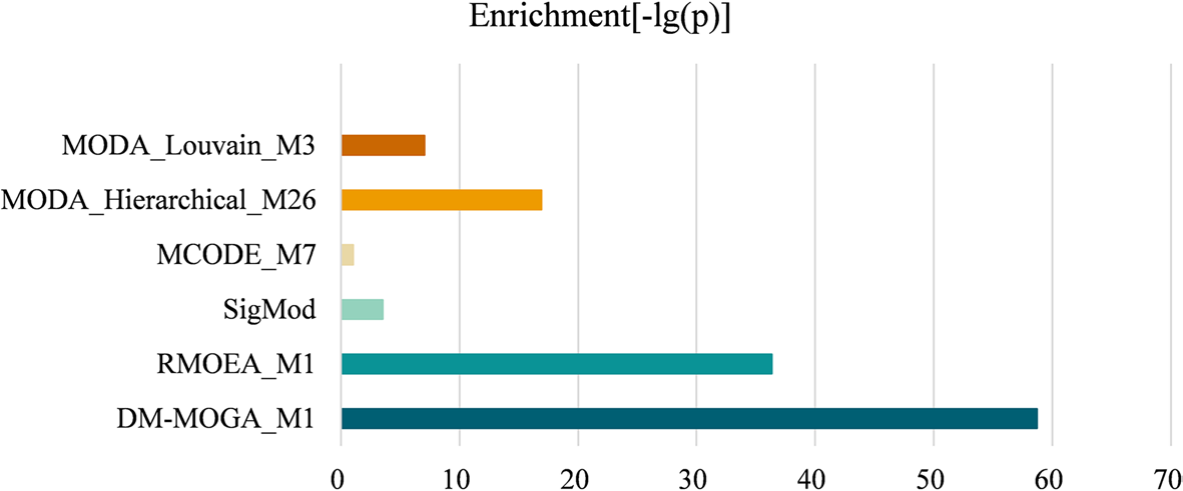
Enrichment of the module discovered by different methods from GSE19804

**Fig. 5:**
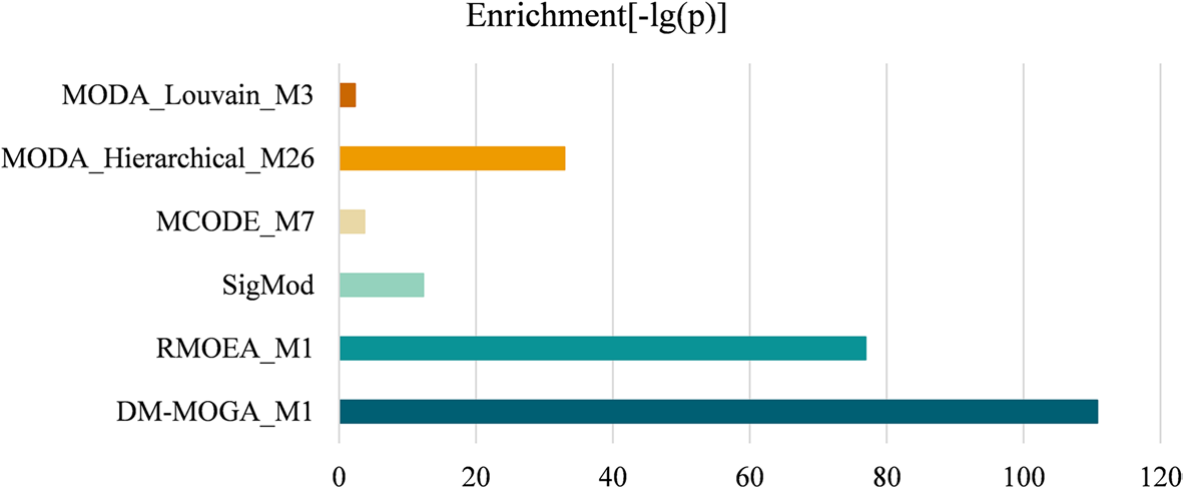
Enrichment of the module discovered by different methods from GSE19188

The quality of the identified module was further quantitatively studied by gene set enrichment analysis [31]. A hypergeometric test is used to estimate the enrichment of the identified module with reference to the ground truth set, where the statistical significance of the enrichment is defined as follows [32]:

8 $$p = \sum\limits_{{k = N_{gm} }}^{{N_{g} }} {\left[ {{{\left( {\begin{array}{*{20}c} {N_{g} } \\ k \\ \end{array} } \right)\left( {\begin{array}{*{20}c} {N - N_{g} } \\ {N_{m} - k} \\ \end{array} } \right)} \mathord{\left/ {\vphantom {{\left( {\begin{array}{*{20}c} {N_{g} } \\ k \\ \end{array} } \right)\left( {\begin{array}{*{20}c} {N - N_{g} } \\ {N_{m} - k} \\ \end{array} } \right)} {\left( {\begin{array}{*{20}c} N \\ {N_{m} } \\ \end{array} } \right)}}} \right. \kern-0pt} {\left( {\begin{array}{*{20}c} N \\ {N_{m} } \\ \end{array} } \right)}}} \right]}$$

where $N_{g}$ is the number of genes in the ground-truth dataset, $N_{m}$ is the number of genes in the identified module, $N_{gm}$ is the number of genes that belong to both the ground truth set and the identified module. A smaller *p* value indicates a more significant enrichment of genes in the identified module. Figures 4 and 5 display the $- \log \left( p \right)$ value of the module obtained by comparison methods running on GSE19804 and GSE19188, respectively. It can be observed that the proposed method obtains significantly better *p* value than the other methods on both two GCNs.

#### Effectiveness of GCMI

**Fig. 6:**
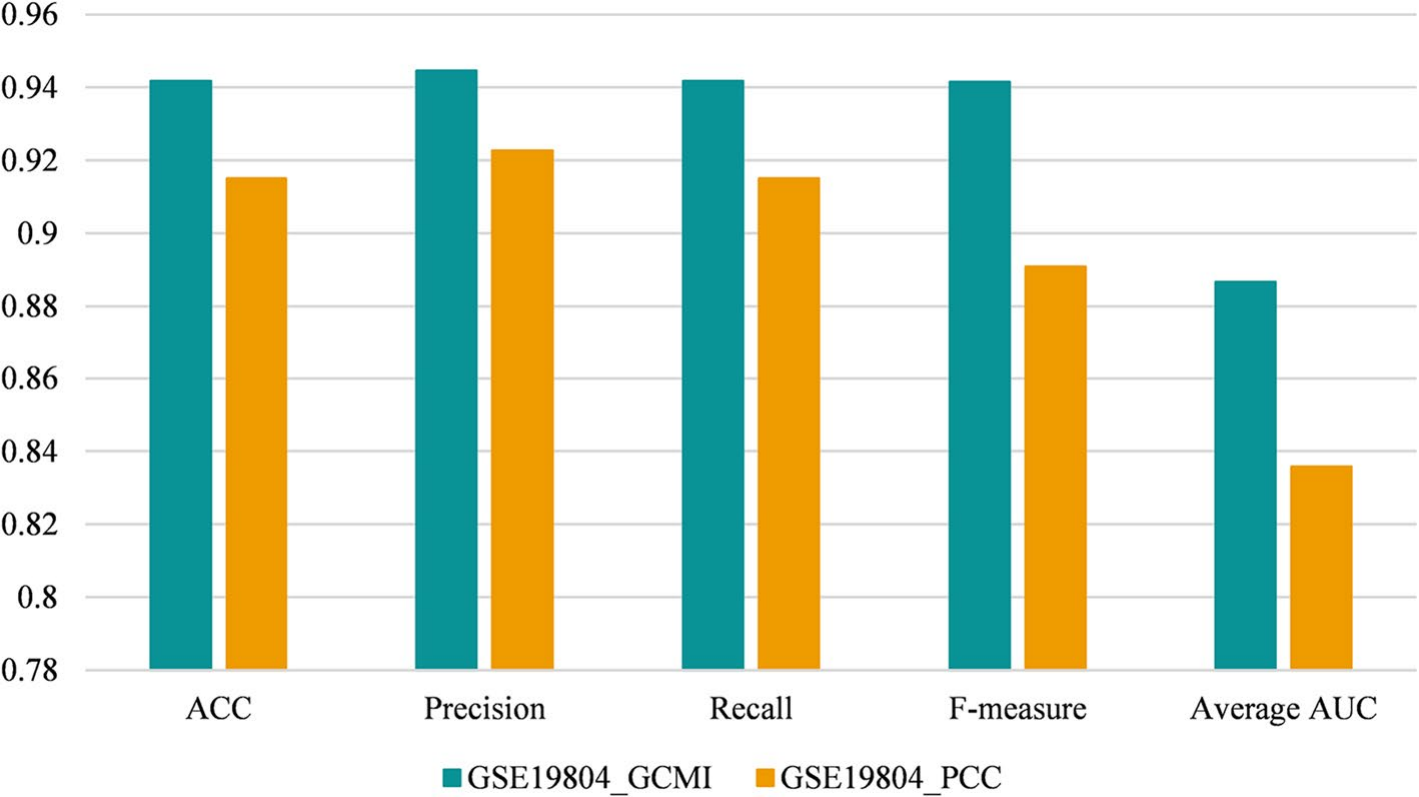
The classification performance of the module identified based on different interaction metrics from GSE19804

**Fig. 7:**
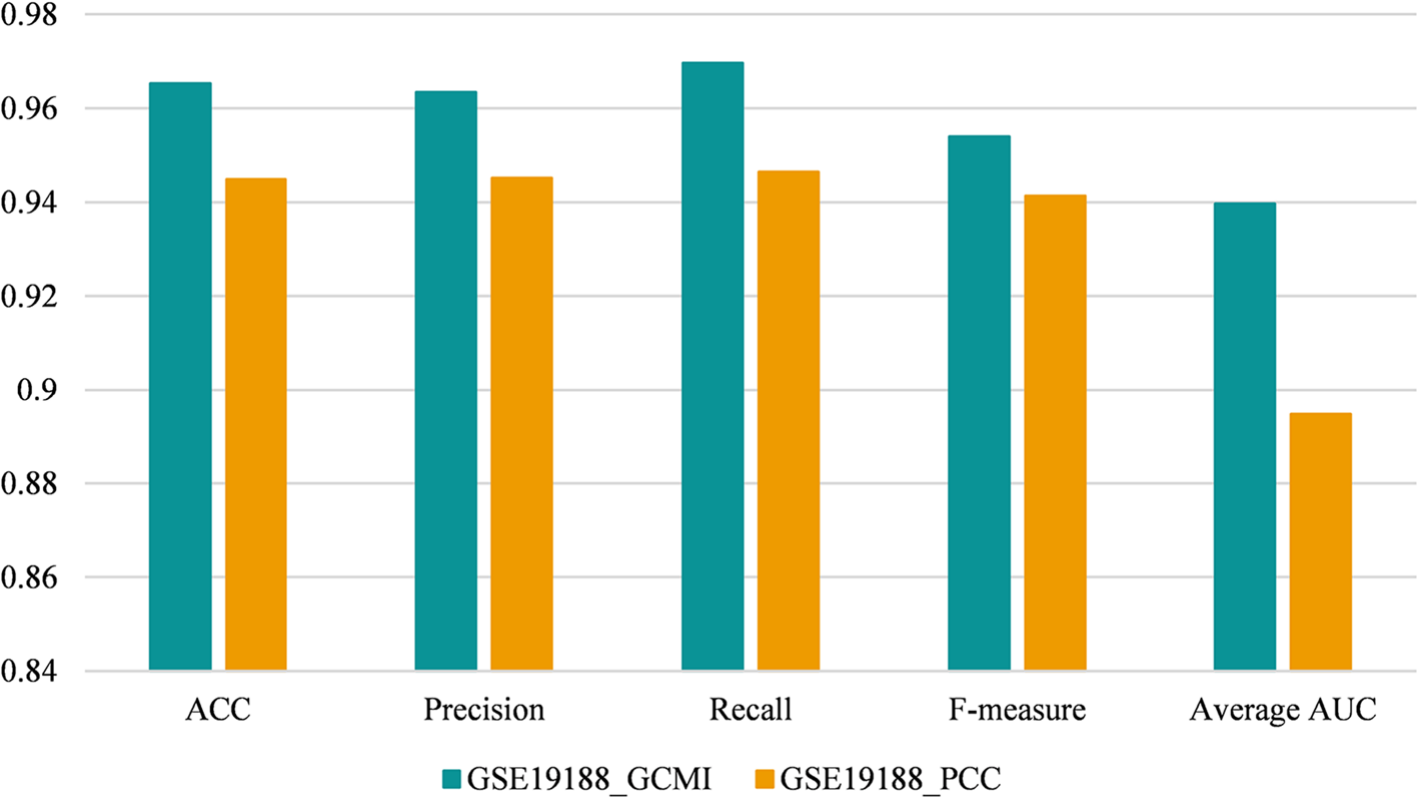
The classification performance of the module identified based on different interaction metrics from GSE19188

In order to study whether the gene–gene interaction metric can affect the efficiency of modules identified by DM-MOGA, we employed the most commonly used Pearson Correlation Coefficient (PCC) to reconstruct GCNs for comparison. Except for the interaction criterion, other steps of network construction remain unchanged. Then the proposed method was applied to detect modules on the new networks. Figures 6 and 7 respectively show the classification performance of the module identified by DM-MOGA based on different criteria on the two datasets. It can be observed that GCMI we choose to calculate edge weights can improve the biological significance of the disease module.

### Identification of modules associated with lung cancer

#### Pathways

**Fig. 8:**
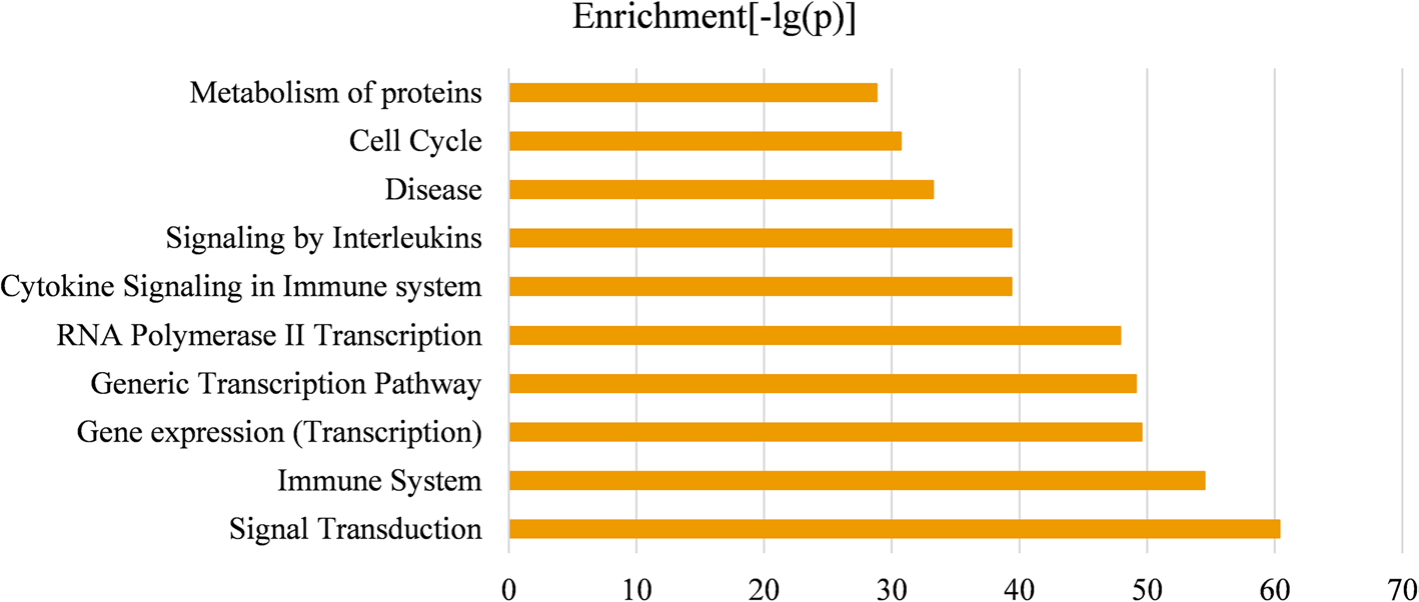
Top 10 significantly enriched pathway terms associated with genes in the identified module of GSE19804

**Fig. 9:**
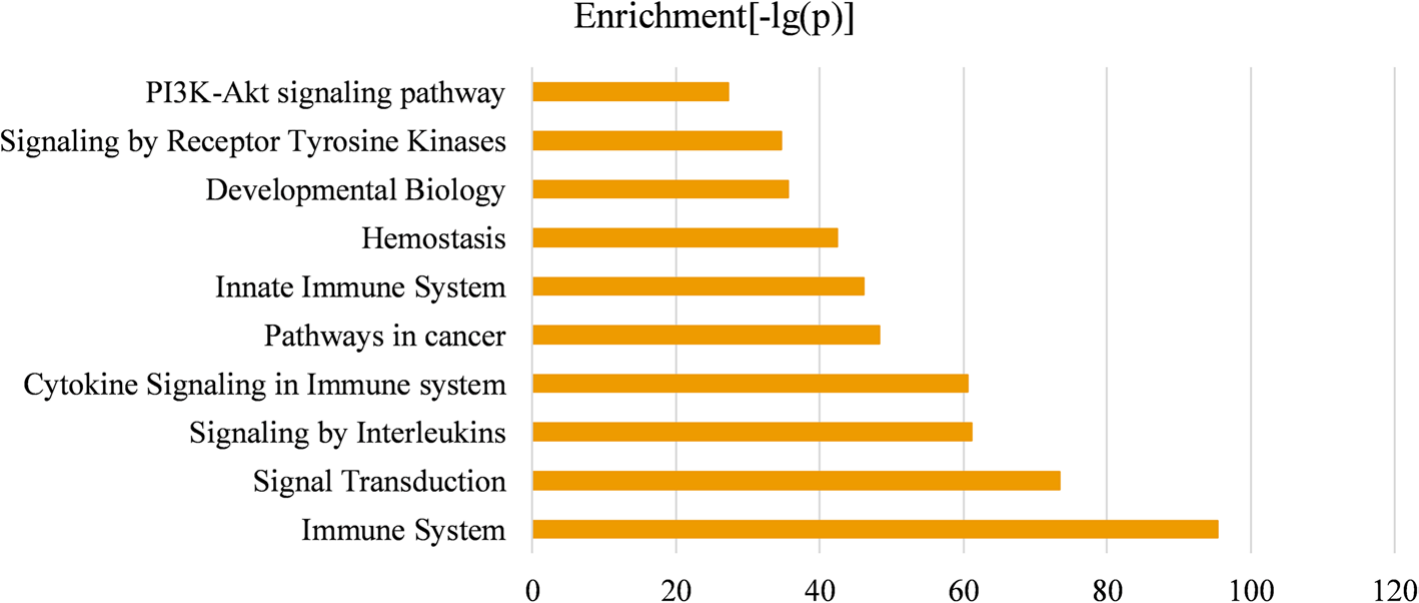
Top 10 significantly enriched pathway terms associated with genes in the identified module of GSE19188

Pathway enrichment analysis was implemented by the KOBAS v3.0 web server, in which four datasets are considered in the analysis, including the KEGG pathway [33], BioCyc, PANTHER, and Reactome [34, 35]. Pathways that are considered to be significant only if the Benjamini–Hochberg adjusted *p* value ≤ 0.05. Pathway enrichment analysis was performed on the disease modules of GSE19804 and GSE19188 independently, and ten pathways with the smallest *p* values ($- \log \left( p \right)$) were respectively displayed in Figs. 8 and 9. Moreover, in Additional file 2, we provided the enrichment significance of these pathways in the two disease modules. Except for the Pathways in cancer pathway that is from the KEGG pathway database, the other pathways in Figs. 8 and 9 are all from the Reactome pathway database [33, 34]. Each pathway was proved to be connected with lung cancer more or less. To be specific, recent studies have found that the mutation frequency of the PTEN locus in lung cancer is high, and there is a strong correlation between loss of PTEN function and positive expression (*p* value < 0.05) of EGFR, TGF-α and P-AKT signal transduction pathway (adjusted *p* value = 4.39E−61) in the development of NSCLC [36]. Besides, there are evidence demonstrating that other signaling pathways enriched in the two disease modules are associated with lung cancer, for instance, the PI3K/Akt signaling pathway (adjusted *p* value = 5.87E−28) inhibiting the metastasis of A549 cell line from lung adenocarcinoma, the receptor tyrosine kinases (RTKs) (adjusted *p* value = 2.1E−35) participating in the signal transmission across the plasma membrane, signaling by interleukin (adjusted *p* value = 4.29E−40) and cell cycle (adjusted *p* value = 1.97E−31) [37–40]. In Figs. 8 and 9, all pathways related to the immune system were confirmed to participate in the progression of NSCLC, including the immune system (adjust *p*-value = 3.23E-55), cytokine signaling in immune system (adjusted *p* value = 4.23E−40) and innate immune systems (adjusted *p* value = 6.65E−47) [41–43] RNA Polymerase II Transcription pathway participates gene expression (transcription) pathway (adjusted *p* value = 2.72E−50) and generic transcription pathway (adjusted *p* value = 7.61E−50). By inhibiting RNA polymerase II-dependent transcription (adjust *p* value = 1.29E−48), cell growth in the malignant cell line A549 can be effectively inhibited [44, 45]. Studies have found that metabolism of protein (adjusted *p* value = 1.53E−29) is related to cancer cachexia. In cancer cachexia, overall protein synthesis is decreasing that is directly proportional to tumor growth [46]. Some researchers believe that cancer is a developmental biology (adjusted *p* value = 2.48E−36) problem. They found that embryos and cancer have a number of common cellular and molecular features [47]. It is known that hemostatic biomarkers (adjusted *p* value = 3.31E−43) can affect the survival and venous thromboembolism (VTE) occurrence in lung cancer patients [48].

#### GO terms

**Fig. 10:**
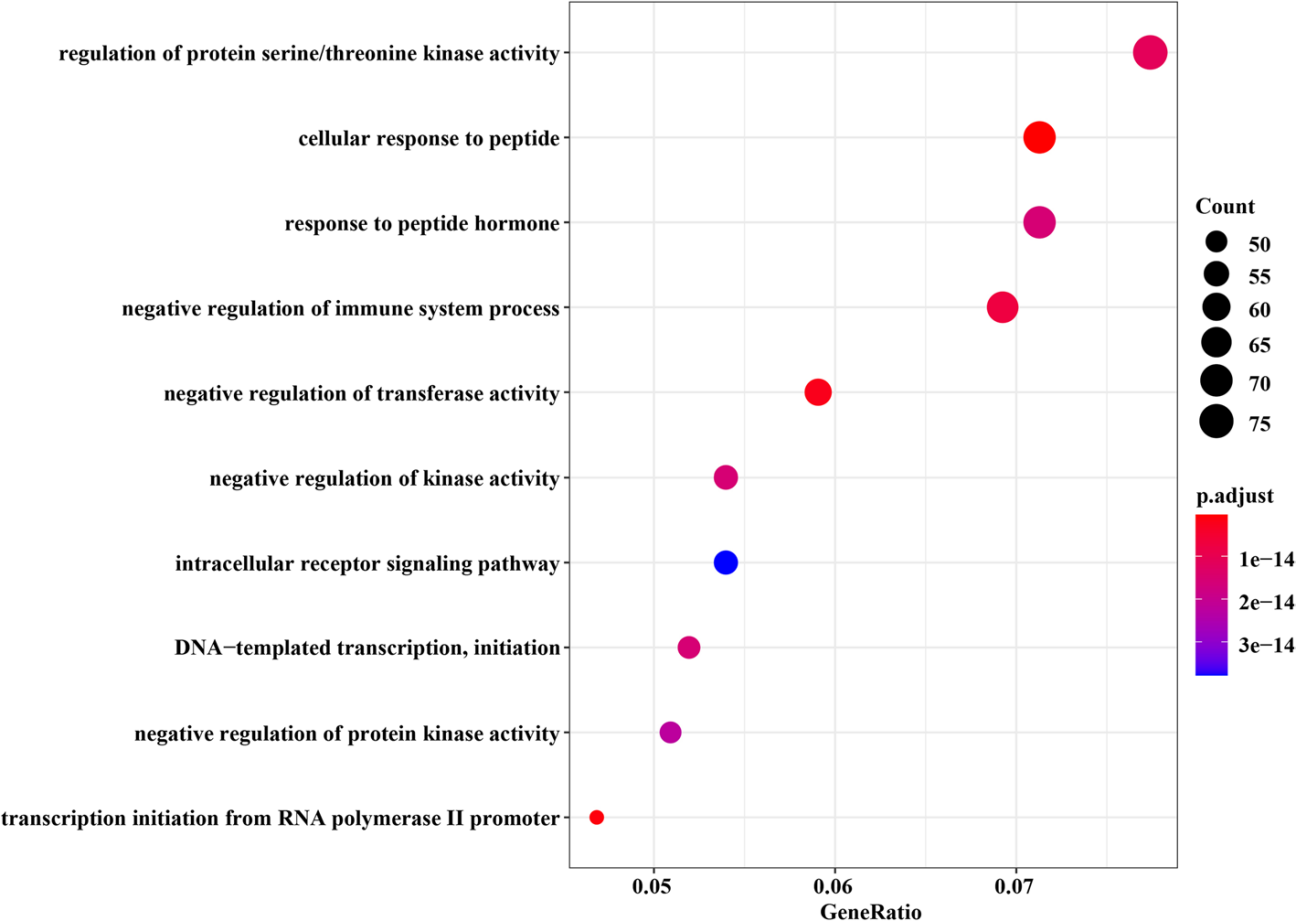
Top 10 significantly enriched GO terms associated with genes in the identified module of GSE19804

**Fig. 11:**
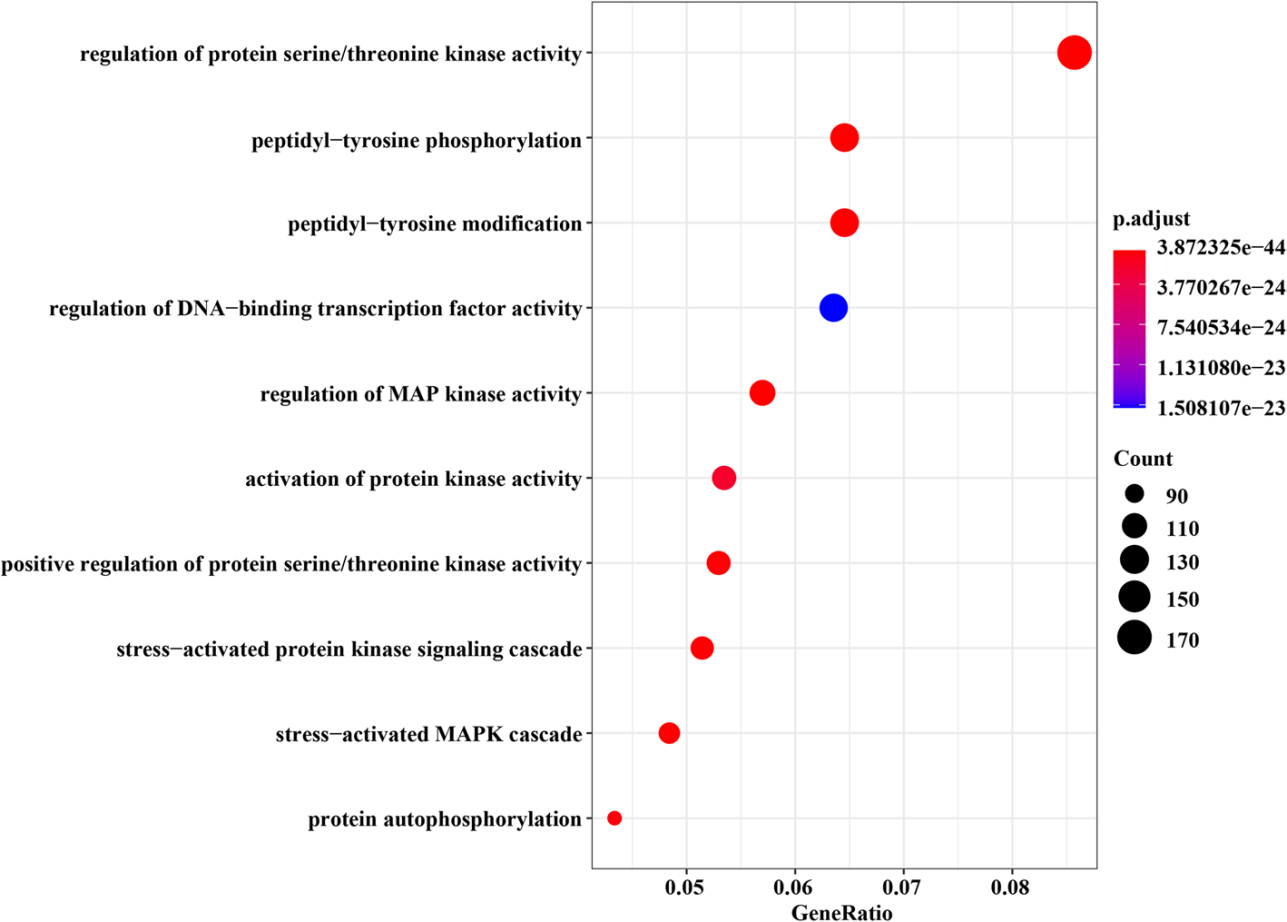
Top 10 significantly enriched GO terms associated with genes in the identified module of GSE19188

GO enrichment analysis was implemented by the R package clusterProfiler [49]. In Figs. 10 and 11, the top ten GO terms that were most significantly enriched in the modules identified from the two datasets are respectively shown. The modules obtained in GSE19804 and GSE19188 were both significantly enriched in regulation of protein serine/threonine kinase activity (GO:0071900, adjusted *p* value = 3.87E−44). Under EGF-stimulated conditions, it is revealed that proteins interacting with B-Raf are enriched in regulation of protein serine/threonine kinase activity, and several interacting partners of B-Raf are enriched in NSCLC [50].

Besides, the core module from GSE19804 was mainly enriched in GO:1901653, GO:0043434, GO:0002683, GO:0051348, GO:0033673, GO:0030522, GO:0006352, GO:0006469, GO:0006367, corresponding to cellular response to peptide, response to peptide hormone, negative regulation of immune system process, negative regulation of transferase activity, negative regulation of kinase activity, intracellular receptor signaling pathway, DNA-templated transcription, initiation, negative regulation of protein kinase activity, transcription initiation from RNA polymerase II promoter. Several studies have confirmed that these GO terms are related to the pathogenesis and development of NSCLC. For instance, studies have found that licorice is a potential NSCLC treatment drug, and the targets of licorice are enriched in the biological process of cell response to peptide (GO:1901653, adjusted *p* value = 3.16E−16) which is an important way to stimulate the acquired immune system [51]. DHX36 plays a role in lung cancer cells by regulating signaling pathways such as response to peptide hormone (GO:0043434, adjusted *p* value = 1.57E−14) [52]. Interleukin-34 (IL-34) is significantly enriched in "negative regulation of immune system processes" (GO:0002683, adjusted *p* value = 7.18E−15) which is highly expressed in primary lung cancer tissues and associated with poor prognosis [53]. Exposure to cigarette smoke (CS) can cause injury to the epithelial cells of the respiratory tract and is considered to be one of the pathogenic factors of lung cancer. DEGs were extracted by comparing BEAS-2B cells (a he human bronchial epithelial cell line) before exposure to CS with after, and they are significantly enriched in the negative regulation of transferase activity (GO:0051348, adjusted *p* value = 2.21E−15) [54]. In addition, negative regulation of kinase activity (GO:0033673, adjusted *p* value = 1.57E−14), intracellular receptor signaling pathway (GO:0030522, adjusted *p* value = 3.78E−14), DNA-templated transcription, initiation (GO:0006352, adjusted *p* value = 1.57E−14), negative regulation of protein kinase activity (GO:0006469, adjusted *p* value = 2.25E−14) and transcription initiation from RNA polymerase II promoter (GO:0006367, adjusted *p* value = 1.10E−15) are the most enriched terms by GO enrichment analysis on key lung cancer-related gene sets that have been reported in other studies [55–59].

In Fig. 11, the identified module was mainly enriched in GO:0018108, GO:0018212, GO:0051090, GO:0043405, GO:0032147, GO:0071902, GO:0031098, GO:0051403, GO:0046777, corresponding to peptidyl-tyrosine phosphorylation, peptidyl-tyrosine modification, regulation of DNA-binding transcription factor activity, regulation of MAP kinase activity, activation of protein kinase activity, positive regulation of protein serine/threonine kinase activity, stress-activated protein kinase signaling cascade, stress-activated MAPK cascade, protein autophosphorylation. These GO terms have a certain correlation with lung cancer. Somatic variants that can be detected in the matched lymph node metastases but not in the primary lung cancer, are termed as LME-SMs genes. They are enriched in GO terms, for instance, peptidyl-tyrosine phosphorylation (GO:0018108, adjusted *p* value = 6.70E−33) and peptidyl-tyrosine modification (GO:0018212, adjusted *p* value = 1.16E−32) [60]. In [61], there are 35 genes that have been reported to be related to lung cancer. They are mainly related to GO terms in biological pathways, such as regulation of DNA-binding transcription factor activity (GO:0051090, adjusted *p* value = 1.51E−23), positive regulation of DNA-binding transcription factor activity, etc. In [62], it is found that cPLA2 is over-expressed in NSCLC cells transformed by oncogenic Ras, and cPLA2 is a well-known substrate of MAP kinase and closely related to the regulation of MAP kinase activity (GO:0043405, adjusted *p* value = 8.96E−32). Stem cell factor (SCF) and its receptor c-kit proto-oncogene are co-expressed in at least 70% of small cell lung cancer tumors and tumor-derived cell lines. The binding of SCF to c-Kit leads to receptor dimerization and activation of protein kinase activity (GO:0032147, adjusted *p* value = 1.62E−24) [63]. In other studies, the rest GO terms are also the most enriched terms in the results of GO enrichment analysis on key lung cancer-related gene sets [64–66].

## Conclusions

In this work, to identify disease modules in GCNs, a multi-objective optimization method DM-MOGA is proposed based on the MOEA framework with decomposition. In DM-MOGA, the first step is to respectively construct the GCN on two NSCLC gene expression datasets, in which GCMI between all genes is calculated and considered as edge weights, and then the edges are filtered by referring to the prior knowledge of the PPI network. Secondly, DM-MOGA is separately executed on two GCNs that searches for disease modules by simultaneously optimizing two novel fitness functions, $DBI$ and $CC^{\prime}$. After the evolution is finished, the Pareto-optimal solution with the largest $W^{\prime}$ is selected as the final result.

To examine the validity of disease modules obtained through the above process, a series of experiments performed. First of all, DM-MOGA was compared with several other module identification methods from the following aspects, specifically, the classification effect of disease and control samples guided by modules, the enrichment of modules in the disease-related gene set, and the validity of the edge weight criterion. Then, the correlation between modules and lung cancer was verified by pathway and GO term enrichment analysis. Experiments proved that the biological meaning of key modules obtained by DM-MOGA was more significant.

The proposed method possesses two main advantages. First, two fitness functions that have never been used for module identification problems are introduced which effectively improve the accuracy of the module in guiding patient classification. Second, the boundary correction strategy is designed for local modules, so that nodes with high correlation strength and low degree can be incorporated into the module. However, there are still some works in this field that can be further studied. On the one hand, it is necessary to develop fitness functions that are more suitable for disease module identification; on the other hand, studying the improvement strategies of EAs can further improve search efficiency.

## Supplementary Information

**Additional** **file 1:** The protein–protein interaction information downloaded from HPRD.

**Additional** **file 2: **The union of the 10 most significantly enriched pathways in the two disease modules and the genes overlapping between these modules.

## Data Availability

The code for DM-MOGA is available at https://github.com/LyanMelrose/DM-MOGA.git. The PPIN is available from HPRD (http://www.hprd.org; see also Additional file 1). Information of genes associated with NSCLC is compiled from the MalaCards database (https://www.malacards.org/). Gene expression dataset GSE19804 and GSE19188 are available from the GEO database (GSE19804: https://www.ncbi.nlm.nih.gov/geo/query/acc.cgi?acc=GSE19804; GSE19188: https://www.ncbi.nlm.nih.gov/geo/query/acc.cgi?acc=GSE19188). All tools used in this work are available from the respective links (the KOBAS v3.0 web server: http://kobas.cbi.pku.edu.cn/kobas3, R package limma: http://bioconductor.org/packages/release/bioc/html/limma.html; R package GOSemSim: http://bioconductor.org/packages/release/bioc/html/GOSemSim.html; R package clusterProfiler: http://bioconductor.org/packages/release/bioc/html/clusterProfiler.html; and R package igraph: https://igraph.org/r/).

## Abbreviations

NSCLC: Non-small cell lung cancer
EA: Evolutionary algorithm
DIAMOnD: DISeAse MOdule Detection algorithm
MCODE: Molecular complex detection
MCL: Markov clustering
GWAS: Genome-Wide Association Study
MOEA: Multi-objective evolutionary algorithm
PPIN: Protein–protein interaction network
GEO: Gene expression omnibus
DEGs: Differentially expressed genes
GCMI: Gaussian copula mutual information
GO: Gene ontology
HPRD: Human Protein Reference Database
LM: Local module
DBI: Davies–Bouldin Index
CC: Clustering coefficient
MOP: Multi-optimization problem
SVM: Support vector machine
PCC: Pearson correlation coefficient
RTKs: Receptor tyrosine kinases
VTE: Venous thromboembolism
IL-34: Interleukin-34
CS: Cigarette smoke
LN: Lymph node
SCF: Stem cell factor
