# Supplementary material for: DM-MOGA: a multi-objective optimization genetic algorithm for identifying disease modules of non-small cell lung cancer
Source: BMC Bioinformatics. 2023 Jan 9;24:13. doi: 10.1186/s12859-023-05136-z (PMC9830734; doi:10.1186/s12859-023-05136-z)
Supplement: Supplementary file 2 — Additional file 2: The union of the 10 most significantly enriched pathways in the two disease modules and the genes overlapping between these modules. [file 12859_2023_5136_MOESM2_ESM.pdf]

1.

Table 1 The unit of the ten pathways with the smallest *p*-value enriched in the two disease modules.

Pathways	Enrichment significance in GSE19804_M1	Genes	GSE19188_M1	Genes
Signal Transduction	60.36	AVPR1B, MYH10, RPN2, AVPR1A, IL1RL1, NOXO1, HDAC3, AKT1, AKT3, CRK	73.38	CRKL, MEN1, DLG1, DLG3, SPR, GPR183, ZFYVE16, PTGER1, PTGER2, PIK3CG
Immune System	54.49	TRIM11, PTGS2, RPN2, IL1RL1, GHR, LAT2, KIR2DS2, HSPA9, HUWE1, AKT1	95.39	RNF14, ATP6V1B1, CRKL, AP1M1, IFNAR1, CD226, LIFR, DLG1, DLG3, TYROBP
Gene expression (Transcription)	49.57	RPN2, ZFPM1, SCG2, AKT1, AKT3, LIFR, HDAC5, CAV1, PSMA1, DNMT1	74.34	EP300, RPN2, SMARCC1, LRPPRC, PSMF1, MEN1, AKT1, HIPK1, AKT3, LIFR
Generic Transcription Pathway	49.12	RPN2, ZFPM1, SCG2, AKT1, AKT3, LIFR, HDAC5, CAV1, PSMA1, THRB	66.95	EP300, RPN2, SMARCC1, LRPPRC, PSMF1, MEN1, AKT1, HIPK1, AKT3, LIFR
RNA Polymerase II Transcription	47.89	RPN2, ZFPM1, SCG2, AKT1, AKT3, LIFR, HDAC5, CAV1, PSMA1, THRB	64.32	EP300, RPN2, SMARCC1, LRPPRC, PSMF1, MEN1, AKT1, HIPK1, AKT3, LIFR
Cytokine Signaling in Immune system	39.37	PTGS2, RPN2, IL1RL1, GHR, HSPA9, AKT1, LIFR, IL27, TYK2, MAP3K8	60.56	BCL2L1, RPN2, IL1RL1, GHR, PDGFRB, PSMF1, SPTB, SPRED1, CD36, AKT1
Signaling by	39.37	PTGS2, RPN2,	61.18	BCL2L1, RPN2,

Interleukins		IL1RL1, HSPA9, AKT1, LIFR, IL27, TYK2, MAP3K8, PIK3CA		IL1RL1, IL1RL2, PDGFRB, PSMF1, SPTB, SPRED1, CD36, AKT1
Disease	33.22	AVPR1B, SLC6A3, RPN2, AVPR1A, SND1, AKT1, AKT3, IKBKG, PSMA1, MYC	63.58	RPN2, CD9, PDGFRB, PSMF1, AP1M1, SPRED1, CD36, AKT1, AKT2, AKT3
Cell Cycle	30.71	HDAC1, LCMT1, RPN2, TUBA8, PSMD9, CLSPN, RBBP7, LIN37, PLK4, PSMD13	37.88	RPN2, EXO1, DYNLL1, PSMF1, PLK4, AKT1, FKBP6, AKT3, KIF2A, TPX2
Metabolism of proteins	28.82	SCFD1, RPN1, RPN2, ZRANB1, NUDT14, TUSC3, CDX2, GANAB, PPARGC1A, IKBKG	43.97	RPN2, ZRANB1, CPM, ADD1, TUSC3, PSMF1, CDX2, MEN1, GATA6, KIF13A
Pathways in cancer	28.81	IL7R, HDAC1, TGFB2, RASGRP4, PTGS2, RASSF1, TPM3, RASGRP2, RASSF5, BIRC2	48.26	BCL2L1, CRKL, PDGFRB, NQO1, AKT1, AKT2, IFNAR1, EGF, RARA, RARB
Innate Immune System	26.81	RPN2, LAT2, KIR2DS2, HUWE1, RNF216, IKBKG, MS4A2, MAP3K8, TYROBP, PIK3CA	46.18	BCL2L1, RPN2, DYNLL1, TIMP2, AHSG, PSMF1, CD33, CD36, EPO, IKBKE
Hemostasis	15.39	HDAC1, RASGRP2, TGFB2, TUBA8, CYB5R1, SRC, ZFPM2, DOCK3, ZFPM1, RAP1A	42.48	TIMP3, ZFPM2, CD36, AKT1, PPIL2, L1CAM, KIF2A, EGF, PECAM1, GYPC
Developmental Biology	25.74	TGM1, RPN2, NRCAM, LYPLA2, AKT1, AKT3, MAFB, LHX2, TYROBP, PSMA1	35.61	MYH10, RPN2, SPAG9, SPTB, CD36, AKT1, AKT2, AKT3, L1CAM, MYL9

Signaling by Receptor Tyrosine Kinases	23.68	NCF1, STMN1, PAG1, GAB2, RAP1A, AKT1, ADAM12, AKT3, CLTA, MAPK11	34.68	FGFRL1, CRKL, PDGFRB, SPRED1, AKT1, AKT2, AKT3, RAPGEF1, EGF, AXL
PI3K-Akt signaling pathway	10.93	IL7R, GHR, PPP2R3A, IFNAR2, AKT1, AKT3, IKBKG, TSC2, THEM4, PRLR	27.23	BCL2L1, GHR, TNXB, AKT1, AKT2, AKT3, EGF, PIK3CG, HSP90AB1, MAGI2

2. Overlap genes between two disease modules

AASDHPPT
AATF
ABCC9
ACSL3
ADD2
ADRB2
AIMP2
AKAP12
AKAP5
AKT1
AKT3
ALDOB
ANGPT1
ANGPTL1
ANK2
ANTXR1
APPL1
ARHGAP29
ARID4A
ATP6V1E1
ATR
AURKA
AURKAIP1
AXL
BCL11A
BCL6
BCL6B
BLK
BRCA1
BRF2

BRIP1
BTG1
BTK
CAMK2B
CAMK2N1
CAMK2N2
CAMTA2
CARD11
CAV2
CBFA2T2
CBFB
CCDC120
CCDC136
CCNA2
CCNB2
CCNH
CD19
CD226
CD247
CD300LF
CD33
CD79A
CDC14A
CDC25C
CDC6
CDK20
CDK7
CDKN2C
CDT1
CDX2
CEP72
CHD4
CHEK1
CHEK2
CLEC4A
CLEC5A
CNP
CPS1
CR2
CREBBP
CRK
CRY1
CRY2
CSTF1

CTTN
CYLD
DAXX
DBF4
DGKA
DHCR24
DLC1
DNMT1
DNMT3B
DOCK3
DOK3
DOK6
DUSP1
DUSP22
DUSP3
DUSP7
DUSP9
E2F1
E2F4
EED
EEF1E1
EFEMP1
EFNB2
EID3
EIF3B
ELF1
ELK3
EPHB3
EPRS
ERCC2
ETS1
ETS2
FAP
FBXO11
FER
FOSB
FOSL2
FOXO1
FOXO4
FYN
FZD9
GAB1
GAB2
GAS6

GATA2
GCM1
GHR
GJA1
GJA5
GLS2
GPS1
GPX7
GRASP
GRB7
GRIN2B
GRM2
GRM5
GTF2H5
GYPC
HABP4
HDAC1
HDAC3
HDAC5
HDAC6
HECW2
HEXIM1
HEXIM2
HIP1R
HMGN1
HMGXB4
HNF4A
HOXA1
HOXB3
HOXB7
HOXC13
HOXD4
HR
HSPA4
HTATIP2
ICAM1
IFT20
IFT57
IGF1R
IKBKAP
IL15
IL15RA
IL1B
IL1RL1

IL27
IL27RA
IL2RA
IL2RB
IL31RA
IL4R
IL7R
ILF3
ING3
IRAK3
ITPKA
JDP2
JMJD1C
JUNB
KCNJ2
KCNJ8
KCNK1
KCNQ5
KCTD17
KHDRBS2
KIF18A
KIT
KL
KLF11
KRT6A
KSR2
LAIR1
LAX1
LCP2
LDHA
LIFR
LIN37
LRRN4
MAD2L1BP
MAGEA1
MAOA
MAP3K8
MAVS
MCL1
MCM10
MCM2
MCM3
MCM6
MCM7

MDFI
MECR
MEX3B
MGAM
MKNK2
MMP12
MNAT1
MOAP1
MPZL1
MRAS
MRC1
MS4A2
MSN
MSX2
MT2A
MYD88
MYH10
MYOD1
NCF1
NCOA1
NCOR1
NDRG1
NFIA
NFKB1
NFYB
NIP7
NOX4
NPAS2
NPPA
NPR3
NR1H2
NR2F1
NR2F2
NR4A1
NR5A2
NRBF2
NSD1
NTRK3
OASL
OIP5
OLR1
PAG1
PAX5
PAX8

PBX1
PCDH7
PDE3B
PDZRN3
PER2
PFDN5
PGM5
PHACTR1
PHACTR3
PIK3R1
PIK3R2
PIK3R3
PILRA
PIP4K2A
PIP4K2C
PLAGL1
PLAUR
PLCG2
PLCL2
PLK4
PNRC2
PODXL2
POU3F1
POU3F2
PPARG
PPARGC1A
PPARGC1B
PPIA
PPM1D
PPP1CA
PPP1R15B
PPP1R9A
PPP2R5D
PPP4C
PRAM1
PRDX2
PRIM1
PRKCH
PRKD1
PRMT2
PSIP1
PSMC1
PSMC4
PSMD4

PTMA
PTPN5
PTPN6
PTPRB
PTRF
RAB15
RABIF
RAF1
RAI2
RALGDS
RAP1A
RAP2A
RAPGEF5
RARS
RASGRP2
RASIP1
RASSF1
RASSF5
RBBP6
RBBP7
RET
RGN
RGS3
RNF216
RNF31
RNF5
RORA
RPN2
RRAS
RUNX2
RUVBL1
RUVBL2
RXRA
SAP18
SATB1
SERBP1
SERPINH1
SGSM3
SH2B3
SH3BP5
SH3GL1
SHC1
SHCBP1
SHMT2

SIGLEC11
SIRPB1
SIT1
SKAP2
SKI
SLC9A2
SLC9A5
SMARCA4
SMG5
SMG6
SMG7
SMYD1
SND1
SNX6
SNX9
SOCS2
SORBS3
SOX18
SOX9
SPN
SPRY4
SRC
SSSCA1
STAT5A
STAT6
SUV39H1
SYNC
TACC1
TAF1
TARS
TEK
TEP1
TERT
TEX11
TGFB1I1
THNSL2
THRA
THRB
TIAM1
TIE1
TIMELESS
TIPIN
TLE1
TLE2

TLE4
TLR1
TLR3
TLR4
TLR8
TLR9
TMEM161A
TMF1
TMSB4X
TP53
TP63
TP73
TRAF6
TRAK2
TREM1
TREM2
TRIM24
TSLP
TUBA1A
TUSC3
TXK
TYROBP
UBE2D3
UNC5CL
UTRN
VIL1
VRK1
VTA1
WNT2
XPA
YTHDC1
ZAP70
ZBED1
ZBTB16
ZBTB2
ZBTB24
ZBTB25
ZBTB32
ZBTB33
ZFPM2
ZNF148
ZNF205
ZNF439
ZNF559

ZNF607
ZNF646
ZNHIT1
ZNHIT3
ZRANB1
